# A Novel Intelligent Computational Approach to Model Epidemiological Trends and Assess the Impact of Non-Pharmacological Interventions for COVID-19

**DOI:** 10.1109/JBHI.2020.3027987

**Published:** 2020-09-30

**Authors:** Jinchang Ren, Yijun Yan, Huimin Zhao, Ping Ma, Jaime Zabalza, Zain Hussain, Shaoming Luo, Qingyun Dai, Sophia Zhao, Aziz Sheikh, Amir Hussain, Huakang Li

**Affiliations:** 1 School of Computer ScienceGuangdong Polytechnic University (GPNU) 47873 Guangzhou 510665 China; 2 Department of Electronic and Electrical EngineeringUniversity of Strathclyde 3527 Glasgow G1 1XW U.K.; 3 School of Computer ScienceGuangdong Polytechnic University (GPNU) 47873 Guangzhou 510665 China; 4 Department of Electronic and Electrical EngineeringUniversity of Strathclyde 3527 Glasgow G1 1XW U.K.; 5 GPNU 47873 Guangzhou 510665 China; 6 School of MedicineUniversity of Edinburgh 3124 Edinburgh EH8 9AG U.K.; 7 Edinburgh Napier University 3121 Edinburgh EH10 5DT U.K.

**Keywords:** COVID-19, pandemic modelling, singular spectral analysis – Gaussian fitting (SSA-GF), non-pharmacological interventions (NPIs), impact evaluation

## Abstract

The novel coronavirus disease 2019 (COVID-19) pandemic has led to a worldwide crisis in public health. It is crucial we understand the epidemiological trends and impact of non-pharmacological interventions (NPIs), such as lockdowns for effective management of the disease and control of its spread. We develop and validate a novel intelligent computational model to predict epidemiological trends of COVID-19, with the model parameters enabling an evaluation of the impact of NPIs. By representing the number of daily confirmed cases (NDCC) as a time-series, we assume that, with or without NPIs, the pattern of the pandemic satisfies a series of Gaussian distributions according to the central limit theorem. The underlying pandemic trend is first extracted using a singular spectral analysis (SSA) technique, which decomposes the NDCC time series into the sum of a small number of independent and interpretable components such as a slow varying trend, oscillatory components and structureless noise. We then use a mixture of Gaussian fitting (GF) to derive a novel predictive model for the SSA extracted NDCC incidence trend, with the overall model termed SSA-GF. Our proposed model is shown to accurately predict the NDCC trend, peak daily cases, the length of the pandemic period, the total confirmed cases and the associated dates of the turning points on the cumulated NDCC curve. Further, the three key model parameters, specifically, the amplitude (*alpha*), mean (*mu*), and standard deviation (*sigma*) are linked to the underlying pandemic patterns, and enable a directly interpretable evaluation of the impact of NPIs, such as strict lockdowns and travel restrictions. The predictive model is validated using available data from China and South Korea, and new predictions are made, partially requiring future validation, for the cases of Italy, Spain, the UK and the USA. Comparative results demonstrate that the introduction of consistent control measures across countries can lead to development of similar parametric models, reflected in particular by relative variations in their underlying *sigma*, *alpha* and *mu* values. The paper concludes with a number of open questions and outlines future research directions.

## I. Introduction

Since the emergence of SARS-CoV-2 and the resulting novel coronavirus disease (COVID-19), reported to the World Health Organization (WHO) in December 2019 from Wuhan, China, it has rapidly spread around the world. On January 30, 2020, the WHO officially declared the epidemic of COVID-19 as a Public Health Emergency of International Concern [1], which was then upgraded to a pandemic on March 11, 2020. As of July 26, 2020, the total number of confirmed cases has exceeded 16.1 million, along with approximately 645.7 k deaths [2]. The USA has the highest number of confirmed cases with over 4.15 million, in comparison to nearly 86 k in China [3]. The number of cases in countries such as the UK, Italy, Spain and Russia have been growing steadily, and at a rapid rate in countries such as Brazil and India [2]. This has resulted in a severe health crisis, public panic, governmental challenge and a potential humanitarian disaster worldwide.

To ensure timely and effective risk management and disaster relief of COVID-19 in this extremely challenging situation, accurate pandemic modelling to estimate outbreak size is crucial, as it can provide invaluable information to health system leaders, policymakers and governments, as well as the WHO, stakeholders and citizens, to ensure adequate planning and arrangements are made [4], [5].

Given the continuously updated data on the number of daily confirmed cases (NDCC) for any country/region, we posit a number of questions: When will the turning point occur (i.e., the NDCC reach the peak and will start to decline, corresponding to R_t_ < 1)? What will the value peak in the NDCC? How long will the pandemic last? And what will be the outbreak size over the entire pandemic period for a particular country/region? Due to several factors and uncertainties, such as locally infected and imported cases, the accuracy and reliability of the collected data and the number of tested cases, the data and the associated pandemic pattern cannot be completely accurate and can be difficult to understand and analyse [6]. Each country/region may adopt different ways to estimate the R_t_ (an average of six different measures used in the UK), and to detect, diagnose and count cases, especially in the first few months. These have led to formidable challenges for modelling the COVID-19 pandemic [7].

In addition, various degrees of non-pharmacological interventions (NPIs) may be introduced across regions and countries [7], [8]. The city of Wuhan in China, with a population of over 11 million, has been in a state of almost complete lock down from January 22, 2020 (though partially lifted from April 8, 2020). Intensive testing and forced self-isolation measures were introduced to trace cases and suppress the spread of disease. Similar measures were also introduced in other parts of China, a country with 1.3 billion people, which remained in a semi-lockdown state for over two months. The approach has been effective in suppressing transmission and reducing the incidence of COVID-19.

In other countries and regions, the impact of the disease has varied considerably. In East Asia and South-East Asia, NPIs seem to have worked effectively, especially for South Korea, Taiwan and Vietnam, due mainly to the early aggressive action and a rigorous “test, trace and isolate” (TTI) strategy being established and enforced [9]. In Europe, Italy, Germany, Spain and the UK *et al.* have all adopted similar lockdown measures and NPIs. However, the UK, Spain and Italy have been more heavily impacted than Germany and France, which could be in part due to the adoption of delayed and less rigorous TTI strategies (TTIs). Here we can ask a further question: how can we assess the effects of introducing NPIs? To answer this, trend modelling of COVID-19 is crucial, as it can not only help us understand the history of the disease but more importantly, can inform future strategies for public health management and control, including crisis and risk mitigation. Due to inconsistent results derived from various models in the literature, it is imperative that new prediction models are investigated and validated [10].

Since the outbreak of COVID-19 in Dec. 2019, a number of models have been developed for predicting the spread of the disease, which has also been termed “SARS-CoV-2” and “2019-nCoV”. The Susceptible-Infectious-Recovered (SIR) and Susceptible-Exposed-Infectious-Recovered (SEIR) [5], [7], [13], [16] models are the most popular, followed by the Bayesian mechanistic model developed by researchers from Imperial College London [15], and the IHME model from the Institute for Health Metrics and Evaluation (IHME) [17]. Other models include: the exponential moving average model [6] for influence analysis of meteorological factors on the transmission and spread of COVID-19; an artificial intelligence (AI)-inspired method [11] for real-time forecasting of the size, length and end time of COVID-19 across China; symmetrical modelling [12] for COVID-19 in mainland China, specifically in the Hubei province; the Auto Regressive Integrated Moving Average (ARIMA) based time series forecasting model [27] for analysing the COVID-19 outbreak and its trends in India; the Gaussian distribution model [28] for a transmission study which uses both forward prediction and backward inference, and the Gaussian distribution model for estimation of the death rate of COVID-19 in real-time [29]. In addition, many researchers have focussed on the evaluation of non-pharmacological interventions. For example, in [30], the effectiveness of travel restrictions and transmission control measures during the first 50 days of COVID-19 in China, from 31/12/2019 to 19/02/2020, was quantitatively analysed and validated, and demonstrated that the control measures potentially averted hundreds of thousands of cases. In [31], the potential effects of social distancing interventions in Singapore was assessed. In [32], a parameterized SEIR model was used to assess the impact of different control measures and identify key factors. All these conventional models rely on various assumptions and have quite a few parameters, which often require different data inputs and focus mainly on one country or region. The predicted results are of high uncertainty and their generalisability to different countries and regions is limited, making it difficult to identify comparisons between trends, especially when trying to account for the impact of complicated and varying NPIs. In this study, we develop a novel model that uses the NDCC only to predict trends in the incidence of COVID-19. We aim to address the challenges identified above through our model, and in particular, to link the overall impact of NPIs, rather than any individual measures, to quantitative parametric models.

## II. The Proposed Method

**Fig. 1.**
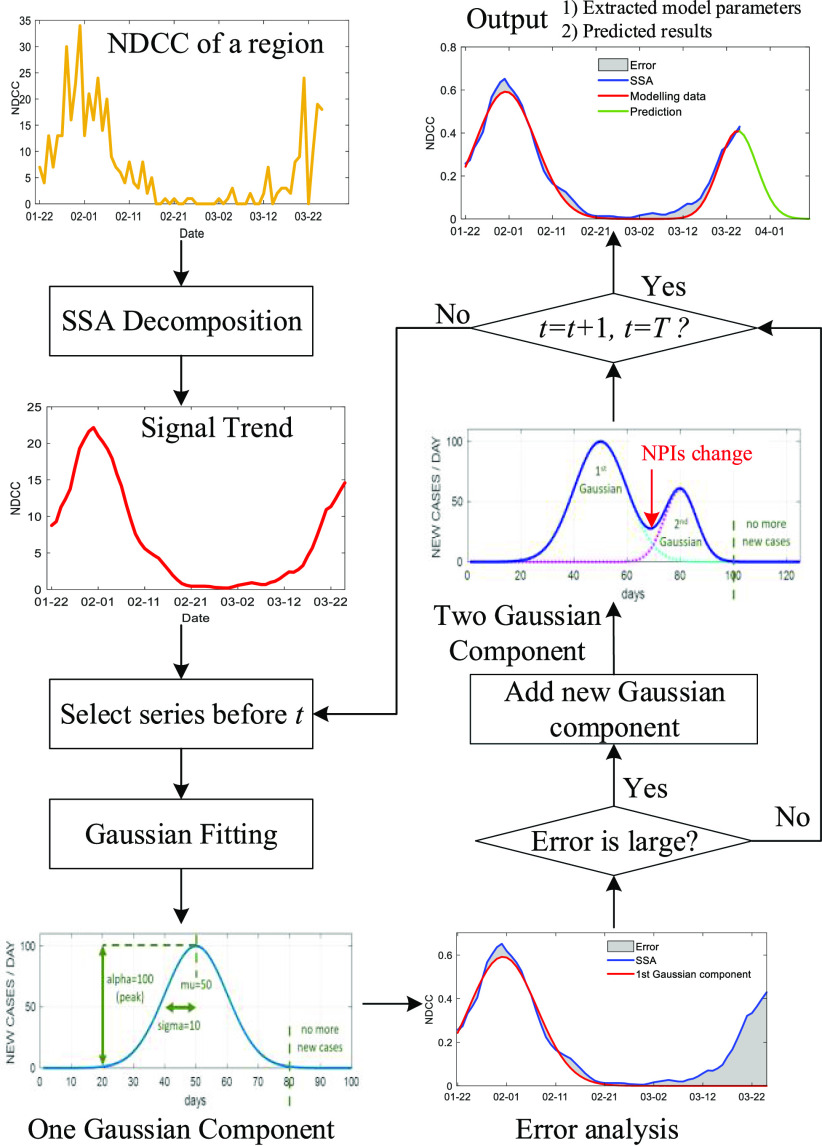
The flowchart of SSA-GF.

An overview of the proposed model is illustrated in Fig. 1, and a detailed description of the implementation and design details of our proposed model is presented as follows.

### A. Procedure

For a given country or region, the NDCC over a certain period is taken as a time series for analysis, without additionally modelling the reproduction numbers (R_0_ or R_t_), daily death rate and daily recovery rate, as done in conventional models. By assuming randomness in the data acquisition, including inaccuracies and cross-region-variations of the data, the NDCC time series can be seen as a stochastic process [14], which, in turn, can help estimate a number of model parameters. Due to the availability of limited and ambiguous observations, a high degree of uncertainty is associated with the accuracy of estimated trends. To simplify the process of data modelling, the singular spectral analysis (SSA) approach [15] is utilised here to extract the overall trend of the signal from this time series. As a nonparametric spectral estimation method, SSA combines different elements from classical time series analysis [18], and applies multivariate statistics to dynamic systems and signal processing. Thus, it has considerable potential for analysing complex random observations [15], and is exploited here for the first time.

The SSA approach can decompose a time series into different components via the singular value decomposition (SVD) [16] of the lag-covariance matrix, rather than frequency domain analysis. For a given 1-D time series, NDCC data, $x\ = [ {{x_1},{x_2}, \ldots,{x_N}} ]{\rm{\ }} \in {\mathbb{R}^N}$, can be embedded in the lagged columns of the matrix X, namely the trajectory matrix, by an embedding a window of size L and lagged factor K = N − L + 1. The matrix X has equal values in the anti-diagonals, and is a Hankel matrix.

\begin{equation*}
X\ = \left({\begin{array}{cc} {\begin{array}{cc} {{x_1}}&{{x_2}}\\ {{x_2}}&{{x_3}} \end{array}}&{\begin{array}{cc} \cdots &{{x_K}}\\ \cdots &{{x_{K + 1}}} \end{array}}\\ {\begin{array}{cc} \vdots & \vdots \\ {{x_L}}&{{x_{L + 1}}} \end{array}}&{\begin{array}{cc} \ddots & \vdots \\ \cdots &{{x_N}} \end{array}} \end{array}} \right) \tag{1}
\end{equation*}

By applying the SVD to the generated trajectory matrix, various singular components can be extracted in accordance with the derived eigenvalues $({{\lambda _1} \geq {\lambda _2} \geq \cdots \geq {\lambda _L}})$ and eigenvectors $({{U_1},{U_2}, \ldots,{U_L}})$. These extracted singular components usually contain varying trends, oscillations of certain periodic components, and noise [15]. Therefore, the trajectory matrix X can be reconstructed as the sum of several components ${X_i}|i \in [ {1,d} ]$

\begin{align*}
&X\ = {X_1}{\rm{\ }} + {X_2} + \cdots + {X_d}\tag{2}\\
&{X_i} = \sqrt {{\lambda _i}} \ {U_i}V_i^T,{\rm{\ }}{V_i} = \frac{{{X^T}{U_i}}}{{\sqrt {{\lambda _i}} }} \tag{3}
\end{align*}

After eigen value grouping and diagonal averaging, a subset of ${X_i}$, containing the main trend component, is selected to project the matrices into a new 1-D signal $x'$. This signal trend from the time series $x$ can be regarded as a deterministic signal rather than a random variable for further analysis. As a result, it can be applied as a model-free tool for smoothing, noise reduction, trend extraction, periodicity detection, and seasonal adjustment [15].

Taking the initial trend signal $x'$ extracted from the SSA as input, *t* is a time series vector with the same size of *x*, a mixture of Gaussian fitting is used to characterise sequential components within $x'$.

\begin{equation*}
x' = \ f\ \left(t \right) = \ \alpha *{e^{\left({ - {{\frac{{\left({t - \mu } \right)}}{{{\sigma ^2}}}}^2}} \right)}}\tag{4}
\end{equation*}

As there are a number of random variations that may affect the observed data, such as technical inadequacy, management inconsistency or political reasons, the reported NDCC is likely to comprise a stochastic component. According to the central limit theorem, the sum of these complicated factors is assume to satisfy a Gaussian distribution, subject to a sufficient number of observations being collected [20].

**Fig. 2.**
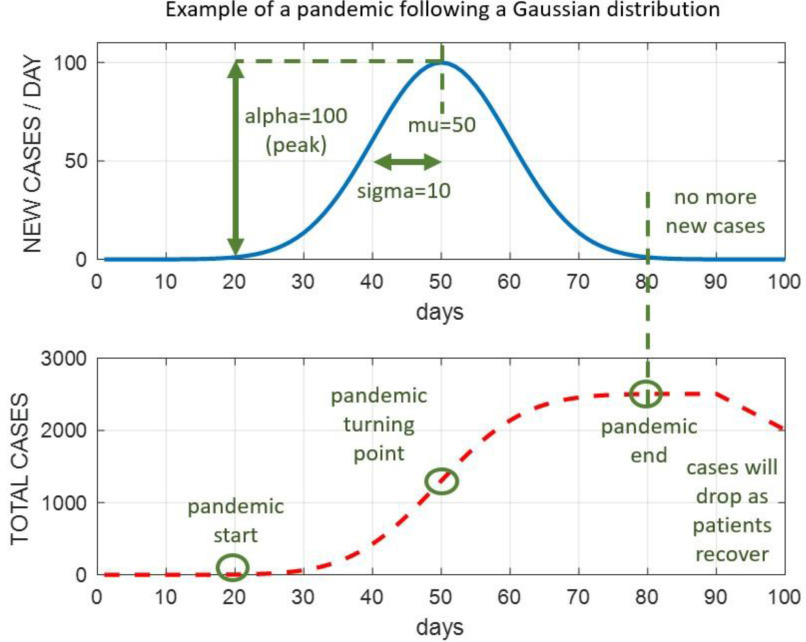
**An example to show the NDCC of a pandemic following a Gaussian distribution with *alpha* = 100, *mu* = 50, *sigma* = 10:** NDCC (top) and cumulated NDCC (bottom), where the pandemic starts from day 20 for 6*sigma = 60 days until day 80, 30 days before and after it reaches the peak in the NDCC curve, namely the date of the turning point day, when the NDCC starts to drop.

Each of the extracted Gaussian components has three key parameters (Fig. 2), the amplitude *alpha (*$\alpha $*)*, the mean value *mu (*$\mu $*)*, and the standard deviation *sigma (*$\sigma $*)*. *Alpha* indicates the height of the curve's peak, which refers to the peaked daily confirmed cases. *Mu* is the central position of the curve, i.e., the date of the turning point when the NDCC starts to drop. The *sigma* links the width of the curve in days, i.e., the total number of days from the start-point to the endpoint of the pandemic, which can be approximated roughly as six times the *sigma* value in days [20]. For a Gaussian curve with a large *alpha*, its value may still be significant after three times the *sigma* value. In this case, we determine a later day, when the number of daily confirmed new cases is no more than a specified threshold, say 3.

**Fig. 3.**
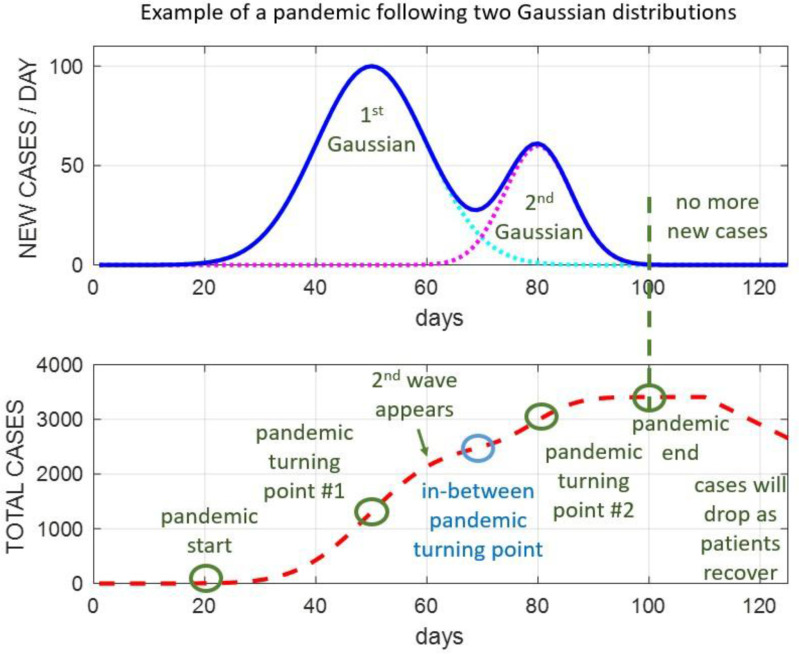
**An example to show the cumulated NDCC of the pandemic following a mixture of two Gaussian distributions:** The pandemic starts from day 20 and lasts until day 100, and experiences three turning points on days 50, 70 and 80, corresponding to a decrease, increase and a further decrease in NDCC trends, as indicated by the NDCC curve in blue.

In contrast to conventional Gaussian mixture models, our proposed SSA-GF is constrained to use up to two Gaussians for extracting the Gaussian components within the SSA trend at any given time. If there is no NPI or with consistent NPIs, only one Gaussian component is required (Fig. 2). If on a certain day, the NPIs are changed, either newly introduced or withdrawn, another new Gaussian component will be activated (Fig. 3), taking effect together with the previous Gaussian component. For this reason, we limit the number of Gaussians to one or two at any given time. On the other hand, for the case of varying NPIs adopted at different times, the total number of Gaussian components spanning a long time period can exceed two, although only up to two are used to overlap with each other.

For a Gaussian component being extracted before the end of the entire period, its parametric model can be used to estimate the values until the end of the period. If the estimated value deviates too far from the extracted SSA trend, a new Gaussian component is introduced. Eq. (4) can then be extended to Eq. (5), where ${t_1}$ and ${t_2}$ are non-overlapped time series vectors, with an accumulated length equal to *N*.

\begin{equation*}
x' = {\alpha _1}{\rm{\ *}} \ {e^{\left({ - {{\frac{{\left({{t_1} - {\mu _1}} \right)}}{{\sigma _1^2}}}^2}} \right)}} + {\alpha _2}{\rm{*}} \ {e^{\left({ - {{\frac{{\left({{t_2} - {\mu _2}} \right)}}{{\sigma _2^2}}}^2}} \right)}}\tag{5}
\end{equation*}

The entire process continues to cope with any newly fed observations to update the derived SSA trend and Gaussian components, in order to further refine the estimation and prediction for future dates.

## III. Comparative Results

We validate our predictive model using retrospective data available from China and South Korea, as the pandemic in these two countries seems to have been successfully suppressed. Next, we estimate pandemic models for the UK, the USA, Italy and Spain in an attempt to predict their future COVID-19 incidence trends.

### A. Data Sources

The data for Italy, Spain, the UK, the USA and South Korea is collected from the Center for Systems Science and Engineering, Johns Hopkins University [2]. For cities and provinces in China (i.e., Beijing, Guangdong, Hubei, Shanghai and Zhejiang), we extracted our data from statistics published by the Chinese authorities [3]. The data collection period, which spanned from January 22, 2020 to March 28, 2020, was used to build and test our pandemic models, and the data which spanned from March 29, 2020 to April 11, 2020 was used for validation. As the daily data reported in [2] and [3] represents accumulated confirmed cases, we differentiate the entire data to obtain the time-series data of the NDCC.

### B. Validated Prediction Results: China

Five cities and provinces were selected from China for analysis, including Beijing, Guangdong, Hubei (Wuhan is the capital city), Shanghai, and Zhejiang. Beijing was selected for its strong links with Wuhan, its large population and as the capital of China. The other three regions were selected as they are geographically close to and have strong economic links with Wuhan and Hubei.

**Fig. 4.**
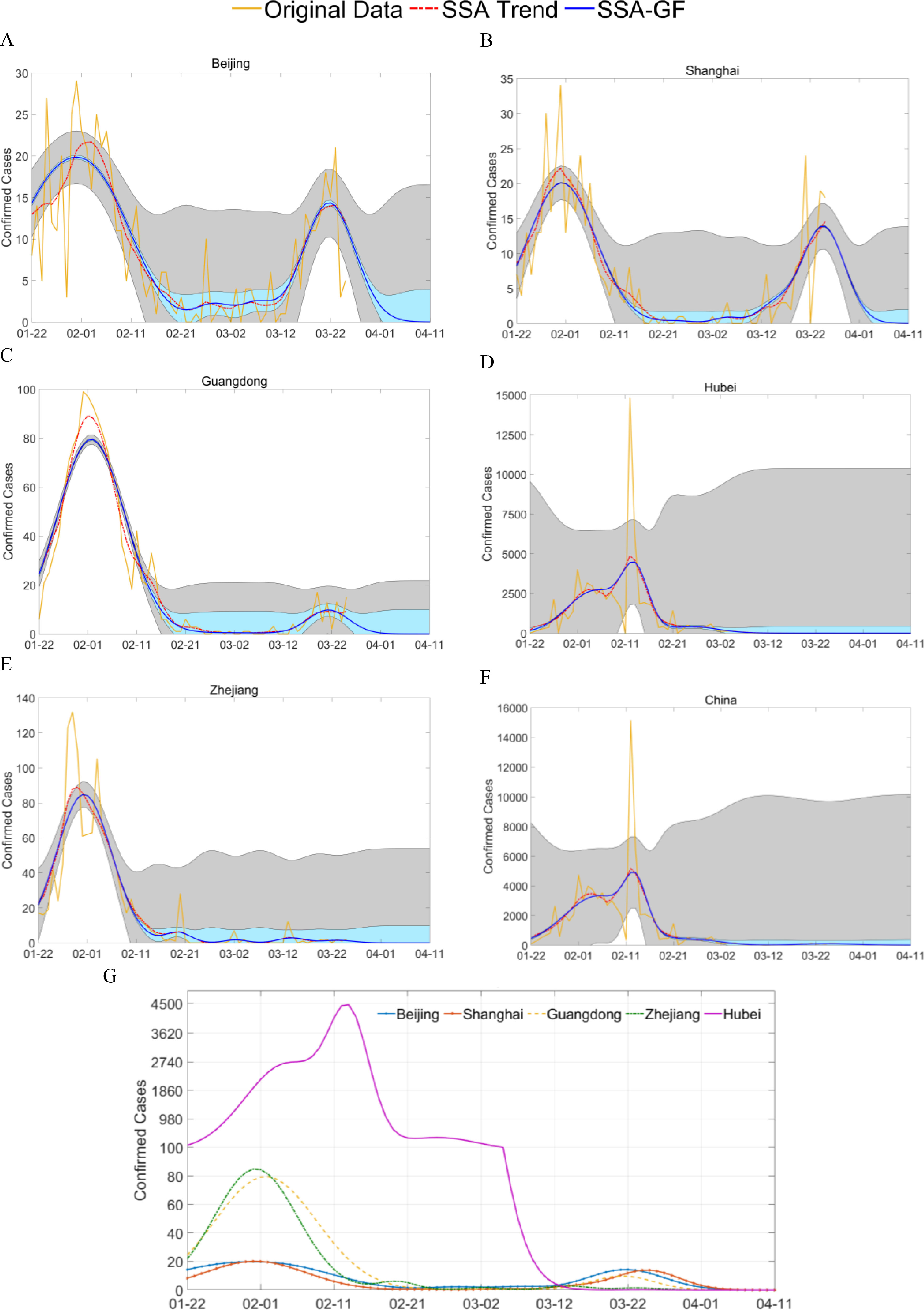
**Time series of daily confirmed cases of COVID-19 from Beijing, Shanghai, Guangdong, Hubei, Zhejiang of China:** (A-F) show results from the five places and all of China, and depict the confirmed cases, the extracted trend from the SSA, and the estimated trend from the extracted SSA-GF model. Shaded areas in blue and grey indicate the estimation errors of the trend from the SSA and the original observation, with a confidence level of 95%. (G) shows the results of the SSA-GF model for the five places, where a nonlinear scale was applied to the data from Hubei to cope with its large data range, when plotting on one graph for comparison.

According to the predicted results, using the data available up to March 28, 2020 (Fig. 4 and Table I), different numbers of Gaussian components were extracted for the NDCC time series, for each of the five places. We selected the Gaussian component with the highest peak value (*alpha*) as the major component, and discarded all those whose peak values were less than 5% of the major peak. For Beijing, there were three noticeable Gaussian components. The first component had a *mu* of January 26, 2020 an *alpha* of 14.26 and a *sigma* value of 7.61, i.e., the corresponding Gaussian component peaked on January 26, 2020 with a height of 14.26. The second component, which was the major one, had a *mu* of February 07, 2020, a *sigma* of 6.96 and an *alpha* value of 16.93, followed by the third component, which had a *mu* of March 23, 2020, a *sigma* value of 5.15 and an *alpha* of 14.39. For the two Gaussian components of Guangdong, the *mu* values of the major one and the following one were February 2 and March 21, 2020, respectively, with *sigma* values of 6.96 and 4.19, and *alpha* values of 79.49 and 9.83, respectively. For Shanghai, the first component was the major one, and had a *mu* of February 1, 2020, a *sigma* of 6.92 and an *alpha* of 20.13. The second Gaussian component had a *mu* of March 25, 2020 and an *alpha* of 13.96.

**TABLE I:** Estimated Endpoint Dates of the Pandemic and the Total Number of Cases for Five Places in China, Including Imported Cases Using the Results in Table I

Cities/Provinces	Beijing	Shanghai	Guangdong	Hubei	Zhejiang
Predicted cases by 28/03/20	584	436	1,402	67,919	1,195
Real values by 28/03/20	573	485	1,467	67,801	1,251
Error (%)	1.92	-10.1	-4.43	0.17	-4.48
Estimated End date	06/04/20	07/04/20	03/04/20	12/03/20	27/02/20
Estimated total cases	627$ \,\pm\, $71	499$ \,\pm\, $43	1,418$ \,\pm\, $329	67,919$ \,\pm\, $5544	1,195$ \,\pm\, $80
Real cases by the date	587	538	1,514	67,781	1,205

For the Hubei province, there were three major Gaussian components, the corresponding *mu* values were February 05, February 13 and February 26, 2020, respectively, with corresponding *sigma* values of 5.78, 2.73 and 5.27, and *alpha* values of 2681.44, 4462.00 and 404.17, respectively. For the two Gaussian components in Zhejiang, the major one peaked on February 1, and the second on February 20, 2020. The corresponding *sigma* values were 5.65 and 2.63, and alpha values of 84.94 and 6.19, respectively.

In addition, using the models determined by the data until March 28, 2020, the estimated dates for Beijing, Guangdong, Hubei, Shanghai and Zhejiang to have no new confirmed cases would be April 6, April 3, March 12, April 7, and February 27, 2020, respectively, with 95% confidence intervals (Table II). The total confirmed cases were estimated to be 627$ \,\pm\, $71, 1,418$ \,\pm\, $329, 67,919$ \,\pm\, $5,544, 499$ \,\pm\, $43 and 1,195$ \,\pm\, $80, as compared to the publicly released cases of 587, 1,514, 67,781, 538 and 1205, respectively. For Hubei, this corresponded to around 0.11% of the population.

**TABLE II:** Extracted Major Gaussian Components From the NDCC Curves of Five Places in China, From 22/01/20 to 28/03/20

Places	Beijing	Shanghai	Guangdong	Hubei	Zhejiang
*Mu* 1	26/01$ \pm 0.0011$	01/02$ \pm 0.0067$	02/02$ \pm 0.0122$	05/02$ \pm 0.0030$	01/02$ \pm 0.0059$
*Alpha* 1	14.26$ \pm 0.0002$	20.13$ \pm 0.0156$	79.49$ \pm 0.1173$	2681.44$ \pm 0.4840$	84.94$ \pm 0.0760$
*Sigma* 1	7.61$ \pm 0.0028$	6.92$ \pm 0.0074$	6.96$ \pm 0.0133$	5.78$ \pm 0.0026$	5.65$ \pm 0.0064$
*Mu* 2	07/02$ \pm 0.0411$	24/03$ \pm 0.0129$	21/03$ \pm 0.0007$	13/02$ \pm 0.0003$	20/02$ \pm 0.0004$
*Alpha* 2	16.93$ \pm 0.0621$	13.96$ \pm 0.0120$	9.83$ \pm 0.0008$	4462.00$ \pm 4.7557$	6.19$ \pm 0.0007$
*Sigma* 2	6.96$ \pm 0.045$	4.36$ \pm 0.0019$	4.19$ \pm 0.0007$	2.73$ \pm 0.0043$	2.63$ \pm 0.0005$
*Mu* 3	22/03$ \pm 0.0017$			26/02$ \pm 0.0000$	
*Alpha* 3	14.39$ \pm 0.0022$			404.17$ \pm 0.0068$	
*Sigma* 3	5.15$ \pm 0.0017$			5.27$ \pm 0.0010$	

### C. Validated Prediction Results: South Korea

**Fig. 5.**
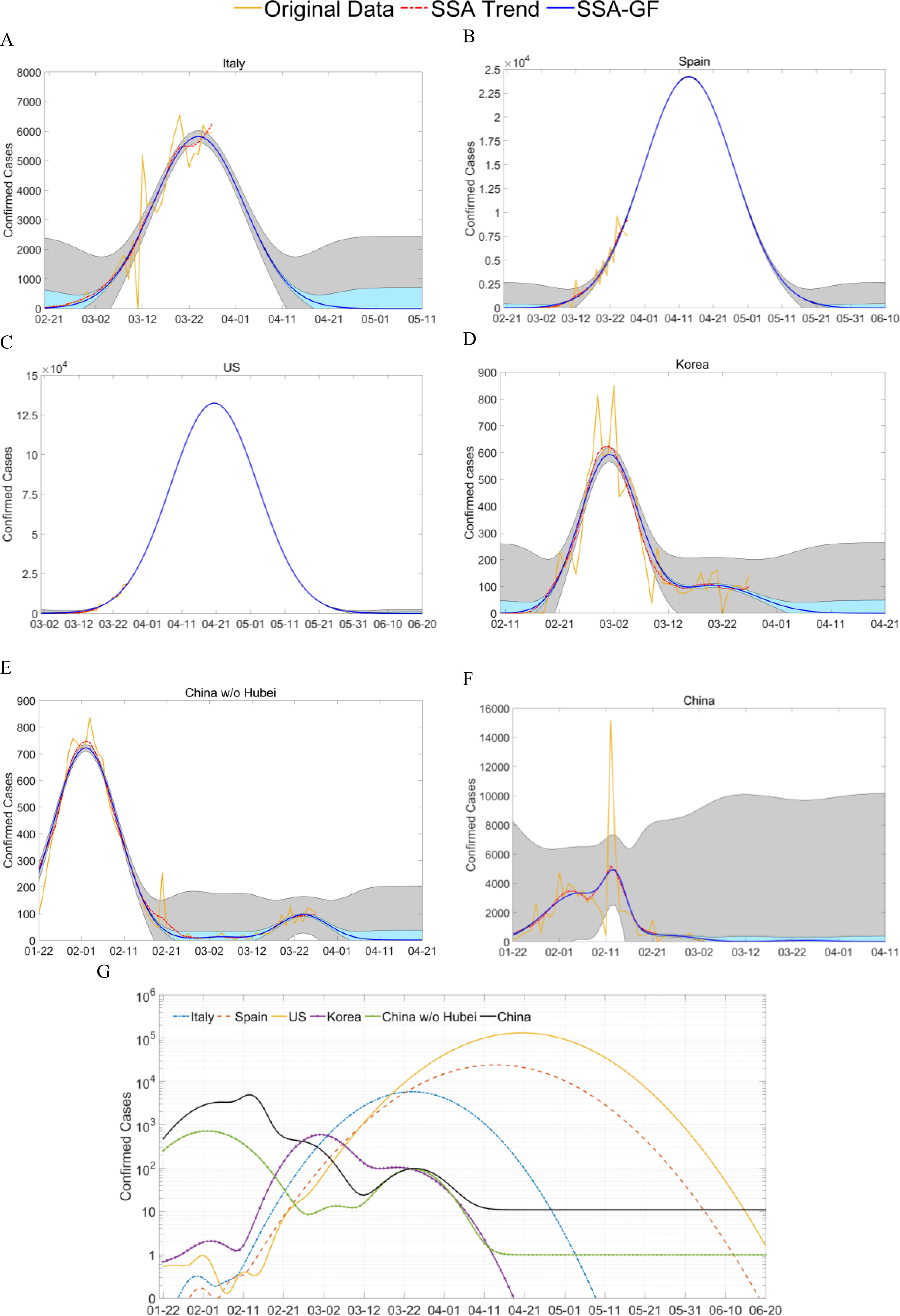
**Daily confirmed cases of COVID-19 from Italy, Spain, the USA, South Korea, China (excluding Hubei) and all of China:** (A-F) show results from the six places, and depict the confirmed cases, the extracted trend from the SSA, and the estimated trend from the extracted SSA-GF model. Shaded areas in blue and grey indicate the estimation errors of the trend from the SSA and the original observation with a confidence level of 95%. (G) shows the results of the SSA-GF model for the five places, where the logarithm scale was applied to cope with the different ranges of data, for comparison.

There were two Gaussian components extracted for South Korea (Fig. 5, Table III), where the major one peaked on March 1 and the following one on March 20, 2020. The corresponding *sigma* values were 5.39 and 7.65 and the peak values (*alpha*) were 590.42 and 103.08, respectively.

**TABLE III:** Extracted Gaussian Components for South Korea, Italy, Spain, the USA and all of China, Excluding Hubei, by 28/03/20, With Different Start Dates. For Italy the Second Peak Was Identified Using Data up to 12/04/20

Country	Italy	Spain	USA	South Korea	China w/o Hubei
Start date	10/02/20	12/02/20	16/02/20	09/02/20	22/01/20
*Mu* 1	23/03$ \pm 0.0011$	13/04$ \pm 0.0067$	20/04$ \pm 0.0122$	01/03$ \pm 0.0030$	02/02$ \pm 0.0025$
*Alpha* 1	5,819.79$ \pm 129.03$	24,228.77$ \pm 90.8346$	132,559.91$ \pm 1,590.56$	590.42$ \pm 0.5568$	723.1801$ \pm 1.1995$
*Sigma* 1	$9.77 \pm 0.0031$	$13.17 \pm 0.0157$	12.75$ \pm 0.2986$	5.39$ \pm 0.0059$	$7.60 \pm 0.0028$
*Mu* 2	14/04$ \pm 0.193$			21/03$ \pm 0.0040$	24/03$ \pm 0.0007$
*Alpha* 2	3938.32$ \pm 236.00$			$103.08 \pm 0.0317$	$96.10 \pm 1.0034$
*Sigma* 2	2.82$ \pm 0.178$			$7.65 \pm 0.0097$	$5.86 \pm 0.0005$

The predicted date to have no new confirmed cases was April 10, 2020 (Table IV). The total confirmed cases were predicted to be 9,953$ \,\pm\, $779, in comparison to the actual number of 10,450 cases, by April 10, 2020, which corresponded to ∼0.02% of the population.

**TABLE IV:** Estimated Endpoint Dates of the Pandemic and the Total Number of Cases for Italy, Spain, the USA, South Korea, and China, Excluding Hubei, Using the Results From Table III

Country	Italy	Spain	USA	South Korea	China w/o Hubei
Estimated by 28/03 /20	91,254	75,168	129,022	9,583	13,782
Real value by 28/03/20	92,472	73,233	122,069	9,450	13,877
Error (%)	-1.32	2.64	5.70	1.41	-0.68
Estimated end dates	01/05/20	09/06/20	18/06/20	10/04/20	08/04/20
Estimated total cases	142,448$ \pm 17,374$	800,034$ \pm 12,843$	4,235,679$ \pm 22,579$	9,952$ \pm 683$	14,351$ \pm 802$
Real cases by the date	207,428	252,372	255,674	10,450	15,006

### D. Predictions for Italy, Spain and USA

For all these three countries, only one Gaussian component was estimated when using the data available until March 28, 2020 (Fig. 5). The *sigma* values were computed as 9.77, 13.17 and 12.75, with *alpha* values of 5,819.8, 24,228.8, and 132, 559.9, respectively (Table III). The dates to have no more than three daily confirmed cases for Italy, Spain and USA were estimated to be May 1, June 9 and June 18, all in 2020, respectively (Table IV). By these dates, the total number of confirmed cases are estimated to be 142,448±17,374, 800,034±12,843, 4,235,679±22,579, respectively, which corresponds to around 0.23%, 1.71% and 1.29% of the population in each of the three countries, respectively. The predicted numbers seem to be under-estimated for Italy yet over-estimated for Spain and the USA in comparison with the official data available until March 28, 2020. This is attributed to the varying NPIs adopted in these countries, as discussed below.

For an NPI based intervention, 2-3 weeks (the incubation period) are normally required to evaluate its impact on infected individuals [16]. As a result, these NPIs have a lagged influence on the NDCC curves. Taking the USA, for example, the total deaths predicted on March 30, 2020 were 100 k-240 k [22], whereas, by using the data available until April 3, 2020, this figure was significantly reduced to 40 k-178 k [23]. This is attributable to the strong NPIs that are known to have been introduced in late March 2020. Similarly, using the data available until April 1, 2020, the total number of cases was estimated to be around 720 k [24] by early May 2020, a significant reduction from the over four million as previously estimated.

Our model can also effectively determine such changes to quantitatively assess the effect of such NPIs at an early stage. By using the most recent data available for modelling, we present updated results in Table V for comparison. For Italy, using the data up to April 12, 2020, a second wave of the infection was identified to peak on April 14, 2020, along with a smaller *alpha* and a much smaller *sigma*. As a result, the total number of estimated cases was increased from 142.4 k$ \,\pm\, \text{17.4}\,{\rm{k}}$ to 175.8 k$ \,\pm\, \text{39.7}\,{\rm{k}}$, in comparison to the actual number of 209,328 cases reported on May 2, 2020. This is attributed to an over-optimistic judgement of the situation and the release of other control measures towards the end of March and the beginning of April. For Spain and the USA, the estimated total numbers of cases were significantly reduced. For Spain, the *sigma* value was reduced from 13.17 to 9.10, and the new Gaussian component was estimated to be 12 days earlier, on April 1, 2020 along with a significantly reduced total number of cases at 209,440±50,519 (in comparison to the actual reported cases of 221,447). This contrasts with the 800 k cases previously predicted on March 28, 2020 (see Table IV). For the USA, the estimated *sigma* value was also reduced from 12.75 to 11.04, and the new Gaussian component was estimated to peak on April 11, 2020 with an estimated *alpha* of 38,104±273. The total number of cases was estimated to be 1,051,890±1,144,278 by May 28, 2020, a significant reduction from over 4.23 million previously predicted (see Table IV).

**TABLE V:** Estimated Endpoint Dates of the Pandemic and the Total Number of Cases for Italy, Spain, and the USA, Using New Data After Introducing Strong Interventions in Spain and the USA, as Reflected by the Much Reduced Sigma Values in Comparison to Those in Table III

Country	Italy	Spain	USA
Data up to date	12/04/20	30/03/20	05/04/20
*Mu*	25/03$ \pm 0.5574$	01/04$ \pm 0.0090$	11/04$ \pm 0.16$
	14/04$ \pm 0.1928$		
*Alpha*	5,845.796$ \pm 129.029$	9,199.422$ \pm 3.6870$	38,103.866$ \pm 272.968$
	3,938.317$ \pm 236.003$		
*Sigma*	9.763$ \pm 0.302$	9.1016$ \pm 0.0048$	11.040$ \pm 0.0724$
	2.821$ \pm 0.178$		
Estimated by the date	157,699	88,439	352,870
Real value by the date	156,363	87,956	337,071
Error (%)	0.85	0.55	4.69
Estimated end dates	02/05/20	07/05/20	28/05/20
Estimated total cases	175,837$ \pm 39,733$	209,440$ \pm 50,519$	1,051,890$ \pm 1,144,278$
Real cases by the date	209,328	221,447	1,730,260

Finally, using more recent data available until June 4, 2020, our model has predicted the total number of cases in Italy, Spain and the USA will increase to 232,874±80,459 by July 10, 252,489±195,285 by July 1, and 2,122,164±295,085 by July 19, 2020, respectively. These higher figures are attributable to the recently announced loosening of NPIs.

### E. Predictions for the UK

We apply our predictive model using data from three different dates to predict pandemic trends at a 95% confidence interval. When using the data available until March 28, 2020, the derived sigma reached a value of 23.51$ \,\pm\, \text{3.77}$, indicating that without NPI measures, over 90% of the population could be infected by the middle of June, 2020. For the prediction using data until April 5, 2020, the sigma was found to be reduced to 11.34$ \,\pm\, \text{0.17}$, where the peak value was estimated to be 5,912.95$ \,\pm\, \text{108.15}$ on April 12, 2020, with the total estimated number of cases: 168,072.03$ \,\pm\, $48,053.93. Finally, the third prediction estimate was obtained using latest data available until May 16, 2020, where four Gaussian components were identified. The peak values were quite similar, which were in the range: 4,930$ \,\pm\, \text{55}$ and 5,346$ \,\pm\, \text{22}$, yet the sigma values were found to vary significantly, in the range: 11.15$ \,\pm\, $0.11 and 23.22$ \,\pm\, $17.34. The most recent Gaussian component was estimated to peak on May 6, 2020 with a sigma of 8.07$ \,\pm\, $0.97. Finally, using data available until June 4, 2020, the total number of cases are now predicted to reach 289,246$ \,\pm\, $164,612 by July 01, 2020, which is 72% more than the number previously estimated using data available until April 5, 2020.

### F. Comparing with other Smoothing Models

In our SSA-GF model, SSA has played a key role in extracting the trend and removing noise, before fitting the Gaussian models. To further validate the efficacy of the SSA in our proposed model, we compare it with three baseline signal smoothing methods, including moving average, Gaussian smoothing and exponential smoothing. Again, we used data until March 28, 2020 for developing and testing our model, and both the modelling and prediction errors for the one week that followed, until April 4, 2020, are given in Table VI and compared with the real values for evaluation.

**TABLE VI:** Comparing SSA with Three Other Signal Smoothing Methods for Italy, Spain, the USA and the UK, using Data Until 04/04/20

Country Real data	Model	Estimated by 28/03/20	Error (%)	Estimated by 04/04/20	Error (%)
USA 121,463 309,699	**Average**	109,487	-10.31	164,012	-47.04%
	**Gaussian**	118,628	-2.82	238,541	-22.98%
	**Exponent**	92,656	-24.1	286,945	-7.35%
	**SSA**	129,022	5.70	404,483	30.61%
Italy 92,469 124,632	**Average**	87,975	-4.86	93,556	-24.93%
	**Gaussian**	90,257	-2.39	103,358	-17.07%
	**Exponent**	74,758	-19.16	107,740	-13.55%
	**SSA**	91,254	-1.32	128,081	2.77%
Spain 73,235 126,168	**Average**	67,879	-7.31	95,967	-23.94%
	**Gaussian**	69,774	-4.73	114,578	-9.19%
	**Exponent**	47,392	-35.29	110,424	-12.48%
	**SSA**	75,168	2.64	172,938	37.07%
UK 26,839 57,198	**Average**	23,202	-13.55	34,629	-39.46%
	**Gaussian**	25,490	-5.03	44,429	-22.32%
	**Exponent**	21,275	-20.73	53,996	-5.60%
	**SSA**	26,594	-0.91	61,906	8.23%

The reason for selecting one week for comparison, was to minimize the effect of model drifting, due to the complicated NPIs adopted in the dynamic process. Note that the window size used for smoothing is 5. In Table VI, negative and positive errors indicate an underestimate and overestimate, respectively. It is evident that SSA gives the lowest modelling error using the data until March 28, 2020, for Italy, Spain and the UK, which indicates the strong capability of SSA in extracting the trend signal of the NDCC, for fitting Gaussians. In terms of data prediction, all other models have underestimated numbers, which indicates their inferior ability in predicting unknown data in the future. The SSA method however, successfully predicted the large increase in the number of cases, subject to no NPIs being introduced. Whilst the predicted values seem to be over-estimated, these can be attributed to the strong NPIs adopted, as explained in detail in the next section.

## IV. Discussion

In this section, the impact of the NPIs are analysed, in accordance with the parameters of the Gaussian components derived from the NDCC curves for China, South Korea, Italy, Spain, the UK and the USA.

### A. Impact of NPIs in China

For Beijing, Guangdong and Shanghai, we can clearly see that the major Gaussian components for all three places have the same shape, reflected by an almost equal *sigma* value of 6.92-6.96 (Table I). This indicates that the same pandemic path was taken for the major Gaussian component in each place, which can be attributed to similar intervention measures being adopted in these regions.

For Hubei and Zhejiang, the derived major Gaussian components were seen to have smaller sigma values for the major components, which can be attributed to early and rigorous NPIs and TTIs adopted in these two places. Their first components had similar *sigma* values, 5.78 and 5.65, which indicated that the pandemic incidence paths in these two places were identical, although Zhejiang had a much smaller number of confirmed cases (Table I). On January 20, 2020, the Health Authority of Zhejiang Province (HAZJ) declared five confirmed cases, since January 17, 2020, all of whom had a travel history to Wuhan [3]. Strict measures were then put in place, and the HAZJ initiated a first-level response to major public health emergencies, on January 23, 2020, which was also put in place in Guangdong on the same day. On January 24, 2020, Shanghai, Beijing and many other cities followed suit, whilst the Hubei Health Authority also upgraded their measures on the same day, from a second-level response announced earlier on January 21, 2020.

The smaller *sigma* values in Hubei and Zhejiang can be attributed to their early stage NPIs, which could effectively alter their existing pandemic paths. An example of this was in Hubei, when diagnosis rules were changed on February 12, 2020, following which the second Gaussian component with an extremely large *alpha* value centred on the next day, indicating a strong NPI. With over 10 k new cases being confirmed, a very large peak was introduced, which led to a much smaller *sigma* value of 2.73 for the newly introduced Gaussian component. A similar yet smaller peak can be found in the pandemic path of Zhejiang on February 20, 2020 with a *sigma* value of 2.63. The comparatively smaller peak can be attributed to an accidental outbreak in a prison, and reflects the stricter measures in Zhejiang compared to other places.

**Fig. 6.**
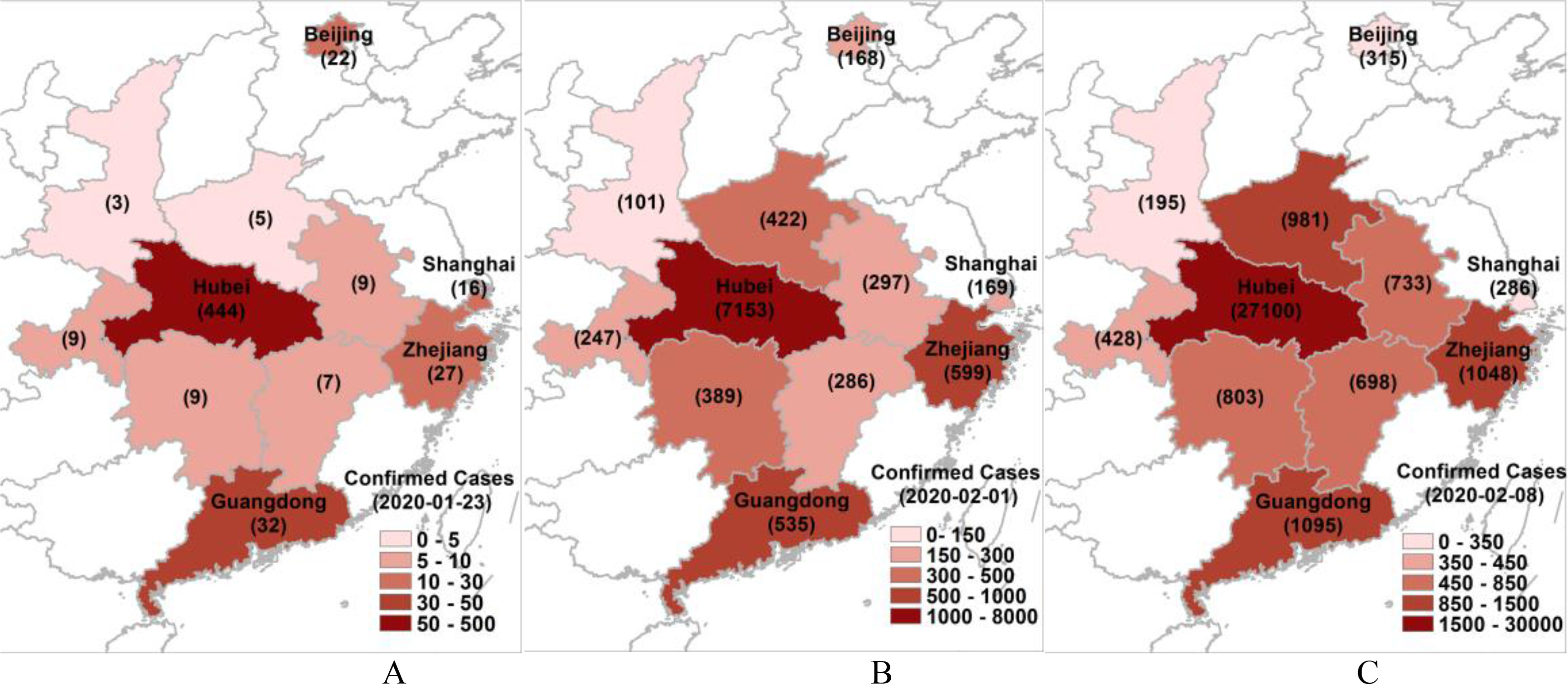
**Confirmed cases of COVID-19 from Beijing, Shanghai, Zhejiang and Guangdong in comparison to Hubei and surrounding provinces of China on January 23 (A), February 1 (B), and February 8 (C), 2020.** (A) shows the number of confirmed cases on January 23, 2020 when Wuhan was locked-down, where Guangdong, Zhejiang, Shanghai and Beijing had the most confirmed cases after Hubei. This indicates their closer links to Hubei and also their rapid response to identify cases when compared to the other six provinces neighbouring Hubei. With strong NPIs, the confirmed cases in (B) in these five places were between 7.64 (Beijing) and 22.19 (Zhejiang) times those in (A), in comparison to 27.44-84.40 times of growth in the other six neighbouring provinces. Comparing (C) and (B), the growth rates in Guangdong, Beijing, Zhejiang and Shanghai were 2.05, 1.88, 1.75, and 1.69, respectively, in comparison to 1.73-2.47 in the other six neighbouring provinces. This indicates the efficacy of similar NPIs, and a higher growth rate of 3.79 in Hubei, due to a poorly uncontrolled situation by February 8, 2020.

The *alpha* values, i.e., the heights of the Gaussian components, especially the major ones in places other than Hubei, were affected by varying degrees of links to Hubei, which included economic, political, educational or societal factors (Fig. 6). On January 23, 2020, whilst Wuhan was in a state of lock-down, the cities of Guangdong, Zhejiang, Shanghai and Beijing, in descending order, were found have the highest number of confirmed cases, more so than any of the six provinces neighbouring Hubei. This indicates that Hubei had very strong links to Guangdong and Zhejiang (especially Wenzhou city), followed by Beijing and Shanghai. This is validated by the corresponding *alpha* values of 84.94, 79.49, 20.13 and 14.26. With effective NPIs and TTIs, the growth rate until February 1, 2020 was within 7.64 to 22.19 times in these five places, in comparison to 27.44-84.40 in the six neighbouring provinces of Hubei. Even with a slightly higher *alpha* value, the pandemic path of Zhejiang peaked on January 31, 2020, two days before Guangdong, which can be attributed to the earlier NPIs. In Shanghai, where similar early action was taken, the first pandemic also peaked on January 31, 2020, whilst in Beijing, the pandemic peaked on February 6, 2020, six days later than Zhejiang and Shanghai.

The extremely high growth rates between January 23 and February 1, 2020 (Fig. 6) in some neighbouring provinces of Hubei can be attributed primarily to insufficient responses. These include the lack of timely monitoring and reporting of confirmed cases, or TTIs, especially in the rural areas of the Henan, Hunan and Jiangxi provinces. Therefore, the growth rates of the confirmed cases in the following week, i.e., from February 1 to February 8, 2020, would be more useful for accurately comparing the effects under the same NPIs and TTIs. During this week, the growth rates of the six neighbouring provinces were between 1.73 to 2.47, whilst the four other places, including Beijing, Guangdong, Shanghai and Zhejiang, had a very comparable growth rate of between 1.69 and 2.05, due to a similar degree of strong NPIs being adopted. On the other hand, a much higher growth rate of 3.79 was evident in Hubei, which indicates a more poorly controlled situation in Wuhan by February 8, 2020, compared to all other parts of China.

It is worth noting that the peaks after the major one in these places can primarily be attributed to imported cases. For Hubei and Zhejiang, since there were no direct international flights during this period, there were no such second peaks in their pandemic paths by March 28, 2020. For Beijing, Guangdong and Shanghai, the daily imported cases peaked on March 23, March 22 and March 25, 2020 with *alpha* values of 14.39, 9.83 and 13.96, respectively (Table I). These were strongly and positively correlated to the flow of international passengers in these three airports. In addition, the *sigma* values for the corresponding three Gaussian components were 5.15, 4.36 and 4.19, smaller than the main peaks, indicating that the in-situ NPIs and measures had effectively suppressed the impact of the imported cases.

### B. Impact of NPIs in South Korea

By adopting early NPIs, including intensive testing, a local lockdown, and effective tracing and isolation, i.e., TTIs, the pandemic path of South Korea was found to be similar to that of Hubei and Zhejiang, where the major Gaussian component was centred on March 1, 2020, with a *sigma* value of 5.39 (Table III), in comparison to 5.78 in Hubei and 5.65 in Zhejiang (Table I).

South Korea also suffered from a second peak primarily due to imported cases imported from abroad, which corresponded to the *mu* of the Gaussian component on March 21, 2020 (Table III). This was similar to the peaked or *mu* values in Beijing, Guangdong and Shanghai occurring on March 23, March 22 and March 25, 2020, respectively (Table I). However, due to less strict measures and interventions compared to those adopted in the Chinese airports, the Gaussian component had a larger *sigma* value of 7.65, in comparison to 5.15, 4.19 and 4.36 for Beijing, Guangdong and Shanghai, respectively (Table I). In addition, the *alpha* value of 103.1 for South Korea was much higher than those of the three Chinese cities which were 14.39, 9.83 and 13.96, respectively. This highlights the significant challenge being posed by imported cases for South Korea.

### C. Impact of NPIs in Italy, Spain and USA

Using data available up to March 28, 2020, it is evident that the less restrictive NPIs in Italy, Spain and the USA, compared to China and South Korea, led to much larger *sigma* values of 9.77, 13.17 and 12.75, respectively. These compare with *sigma* values of 5.65 and 6.96 for China, and 5.39 for South Korea (Table I, Table III). The *sigma* value for Italy was ∼75% less than that of of Spain and the USA, as it had relatively earlier and stricter NPIs. Hence, the predicted total confirmed cases in Italy was ∼142.4 k, far less than than those estimated for Spain, at ∼800 k and the USA, at 4.26 million, when using data available until March 28, 2020 (Table IV). As of March 29, 2020, over 678 k individuals were reported to have been infected worldwide, and more than 31 k people died [2], resulting in an estimated death rate of around 4.57%. Even at 80% of this death rate, the potential death toll in the USA could be over 152 k, which is consistent with the estimated figure of 100 k-240 k from the White House [22].

Both Spain and the USA have adopted strong NPIs since the middle of March, 2020. In the USA, these included a travel ban to European countries, Canada and Mexico, with effect from March 14, March 17 and March 20, 2020 respectively, and a “do not travel” advisory taking effect on March 19, 2020. This was followed by the closure of non-essential businesses in several key states such as New York and California, taking effect from March 21, 2020. In Spain, following the closure of bars, pubs and restaurants *et al.* in Madrid, on March 13, 2020, a state-alarm was issued on March 14, 2020, for 15 days, which was further extended to April 26, 2020. On March 28, 2020, all non-essential activities were halted, along with the ceasing of non-essential business on March 30, 2020.

Comparing the predicted results in Tables IV and V, it is evident that the introduction of strong NPIs has potently reduced 73.8% of cases in Spain and 75.2% of those in the USA. The ban on international travel alone seems to be inadequate, if it is not accompanied with effective tracing, self-isolation and large scale testing, as demonstrated for the cases of China and South Korea [8]. Ceasing of non-essential business also plays a key interventional role, since communal travels and gathering are significantly reduced [7]. Note that our predictions are based on the assumption that existing measures will be in place, which implies the results need to be adjusted if measures are loosened or tightened. The 24% increase in cases from the predicted values on March 28 and April 12, 2020, for the case of Italy, have shown the associated risks of terminating lockdowns in their early stages.

### D. Impact of NPIs in the UK

The initial predicted picture of over 90% of the population being affected, based on the data available until March 28, 2020, is due to the lack of strict measures in the early stages of the pandemic, as reflected by the extremely large sigma value. The predicted figure is consistent with the prediction made in the ninth report by Imperial College London [16], where the peak day of deaths was estimated to be around June 15, 2020. Although the UK progressively introduced lockdown measures since the last week of March, the effects of these are usually observed after 1-2 weeks. Our model's predictions, using data available until April 5, 2020, reflects the impact of these measures. Unsurprisingly, the sigma is dramatically reduced to 11.34$ \,\pm\, \text{0.17}$, and the total number of predicted cases is also significantly reduced to 168,072$ \,\pm\, $48,054.

The lockdown in the UK has shown promising signs in successfully suppressing COVID-19. However, certain control measures have recently been lifted for England, Wales and Northern Ireland. The effect of loosening such measures has been assessed, by developing our predictive model using data available until May 18, 2020, and comparing the results predicted earlier using data available until May 11 and April 5, 2020. The new estimate for the total number of cases is predicted to be 244,615.35$ \,\pm\, $131,228.85 by July 14, 2020, which is 45% more than the predicted cases for April 5, 2020 (168,072.03$ \,\pm\, $48,053.93). This forecast indicates a less than optimistic situation for the incidence of COVID-19 in the UK, on account of its recent loosening of control measures.

## V. Summary and Open Questions

Mathematical simulation models have played a major role in the world's response to COVID-19 [25]. Preliminary results from our proposed SSA-GF based intelligent computational model have clearly demonstrated the efficacy of exploiting SSA with mixture Gaussian fitting models to further enhance our understanding of the pandemic paths of COVID-19. The model has been validated using retrospective data available from China and South Korea, and partially validated by currently available data from Italy, Spain, USA and the UK. The three model parameters, *sigma*, *mu* and *alpha*, are linked to physical meanings and interpretations related to the COVID-19 pandemic. The *sigma* here is shown to directly determine the pandemic path, where a smaller *sigma* value tends to lead to a relatively smaller number of total confirmed cases. By introducing strict NPIs as early as possible, such as physical distancing and TTI measures, the spread of COVID-19 can be suppressed, as reflected by reduced *sigma* and *alpha* values.

There are a number of limitations of our approach. Firstly, as the pandemic is a dynamic process, its incidence trends may continue to vary due to changes in adopted NPIs and other associated factors such as imported cases. To this end, regular dynamic modelling is essential, where updates can be linked to relevant NPIs and other factors [16], [17]. Secondly, the efficacy of the model relies on the accuracy of reported data, specifically the NDCC, which can be under-estimated due to two reasons. One is lack of required knowledge of the disease at the beginning of the pandemic, and the other is lack of sufficient resources, including facilities, medical staff and funds required for testing and analysis. Consequently, the model parameters can be biased by these factors. Thirdly, an accurate model can only be estimated when the number of observations reach a certain threshold, usually after a period of two *sigma* days from the start date of the pandemic.

Based on lessons learnt from this pilot study, we urge the adoption of essential and strict NPIs and TTIs to suppress the spread of COVID-19, especially for high-risk countries and regions. Meanwhile, we continue to apply our model to assess the pandemic situation in other countries, with aims to inform control and risk management policies and practices. Furthermore, we are extending our predictive model to address a number of outstanding COVID-19 challenges, such as estimating the real number of infected cases from total number of confirmed cases and other information, determining optimal values of *alpha* and *mu* of the extracted Gaussian components, and applying our model to analyse the mortality rate and daily reproduction number of the disease. Current work is also exploring potentially complementary insights from social media analytics [33] to enhance the predictive and interpretive capability of our proposed model.

In the future, we plan to further optimize the predictive decision-making capabilities of our intelligent computational model, by exploring its contextual integration with deep machine learning [26], [34], [35], (including generalized zero-shot learning [36]) and probabilistic linguistic information-based approaches [37]. Finally, our model needs to be validated with additional up to date data from a range of countries for more comprehensive evaluation in relation to other state-of-the-art models. This could lead to the development of a standardised predictive model as a potential benchmark tool for decision makers to guide near real-time COVID-19 control and risk management.

## VI. Additional Information

### A. Contributors

JR and HZ conceived and designed the study. YY, PM, SZ and HL collected the data. JR, HZ, AH, YY and JZ carried out the analysis. JR, PM, QD, SL, ZH, AH and AS interpreted the results. JR, AH, YY, PM, JZ and ZH drafted the Article. All authors contributed to the writing of the final version of the Article.

### B. Declaration of Interests

AS is a member of the Scottish Government's COVID-19 Chief Medical Officer's Advisory Group. The view in this article does not represent the views of the Scottish Government.

### C. Data Sharing

All data used are publicly available, and sources are cited throughout.
